# Supplementary material for: Pharmacokinetics and Tissue Distribution of Coumarins from Tagetes lucida in an LPS-Induced Neuroinflammation Model
Source: Plants (Basel). 2022 Oct 22;11(21):2805. doi: 10.3390/plants11212805 (PMC9656319; doi:10.3390/plants11212805)
Supplement: Supplementary file 1 [file plants-11-02805-s001.zip › plants-1974879-supplementary.pdf]

Table S1. Stability test of PE**,** ES, DF, HR, and PU in plasma and tissues matrices.

Nominal concentrations (μg/mL)		30.0						3.0						0.3					
Matrix	Analytes	Autosampler		Long-Term		Freeze-Thaw		Autosampler		Long-Term		Freeze-Thaw		Autosampler		Long-Term		Freeze-Thaw	
		RE (%)	RSD (%)	RE (%)	RSD (%)	RE (%)	RSD (%)	RE (%)	RSD (%)	RE (%)	RSD (%)	RE (%)	RSD (%)	RE (%)	RSD (%)	RE (%)	RSD (%)	RE (%)	RSD (%)
Plasma	PE	8.22	3.33	-5.20	4.14	-5.55	3.19	11.12	1.75	-4.44	0.14	-2.01	0.02	-4.00	7.11	-6.86	2.04	2.80	3.14
	SC	4.74	3.06	-8.41	0.4	-0.10	4.63	8.27	4.44	-14.30	3.23	2.70	1.75	10.18	3.67	-3.50	3.42	-14.02	2.78
	DF	4.21	1.83	-3.40	1.51	0.49	4.29	-1.38	3.34	-3.13	1.20	2.47	0.56	2.59	3.62	-6.74	3.52	-12.33	3.60
	HR	14.32	3.84	3.55	0.85	-5.47	3.27	12.83	2.62	3.99	1.26	-7.90	0.12	8.34	3.82	9.39	2.22	0.67	2.99
	PU	-9.86	0.32	-4.81	0.79	-5.10	3.30	-11.68	2.19	-10.56	3.79	-9.78	0.06	-6.02	6.04	-8.22	3.78	-9.54	2.90
Brain	PE	4.05	2.41	-3.82	7.36	-1.25	1.84	-0.34	0.42	-3.44	2.93	-2.34	0.81	-2.37	2.25	-0.63	2.20	-1.15	2.22
	SC	4.78	0.82	-4.75	5.16	-2.00	2.06	0.37	0.55	-0.94	3.18	-3.09	1.05	-2.15	6.74	-0.19	7.54	-1.98	3.52
	DF	3.21	1.84	-4.35	4.84	-0.35	2.71	3.73	4.47	0.46	7.28	-6.72	6.02	-4.07	3.93	-0.62	4.73	0.03	6.75
	HR	3.86	4.66	-4.72	7.56	-1.02	2.32	2.31	3.40	-0.98	4.02	-5.14	2.86	-3.47	2.28	-0.21	4.32	-0.69	3.43
	PU	4.57	1.36	-4.61	4.89	-1.74	3.57	1.80	6.54	-5.42	7.69	-4.49	1.81	2.13	7.31	3.45	5.33	-6.54	1.62
Kidney	PE	1.61	4.83	7.34	2.86	1.12	0.67	3.99	3.25	2.69	2.61	0.91	1.11	7.06	3.54	3.02	4.06	2.26	1.48
	SC	-0.35	3.02	4.36	0.96	0.49	1.25	5.72	4.22	2.63	4.94	1.51	1.10	7.01	2.06	3.92	2.74	2.62	1.16
	DF	0.66	2.66	5.67	1.09	0.47	2.83	4.79	3.43	8.12	6.10	0.82	2.74	5.83	0.91	5.19	0.61	-0.65	4.96
	HR	1.35	3.92	3.32	3.31	2.25	0.33	2.81	4.89	3.06	2.88	-0.62	2.98	8.17	0.69	5.12	4.41	2.03	0.52
	PU	1.66	5.07	6.46	2.54	-1.02	1.16	5.21	2.66	8.68	4.53	2.01	7.60	7.10	3.77	5.54	2.78	3.61	6.04
Spleen	PE	-1.02	3.87	2.78	1.68	2.38	1.95	-10.42	4.70	3.67	5.55	4.42	9.54	-5.98	4.42	1.51	14.11	2.50	9.85
	SC	-0.99	1.97	-2.12	9.82	-4.22	1.60	-6.71	2.83	2.22	9.10	-2.65	5.26	-7.70	5.57	-9.67	2.35	-9.57	5.92
	DF	1.50	6.07	7.60	7.81	-1.06	3.27	-1.66	9.62	7.09	4.06	0.49	2.05	6.88	8.79	5.43	6.32	-0.67	7.82
	HR	1.18	4.27	2.92	3.61	0.49	2.02	-0.16	11.83	7.44	2.80	4.87	3.30	5.46	6.29	0.22	5.85	-0.63	3.77
	PU	-2.63	5.87	4.26	1.51	2.27	5.02	-4.98	5.34	7.56	4.95	2.88	4.42	-0.09	1.99	-6.92	3.30	-8.53	6.45

PE = 7-*O*-prenylscopoletin, SC = scoparone, DF = dimethylfraxetin, HR = herniarin, and PU = 7-*O*-prenylumbelliferone

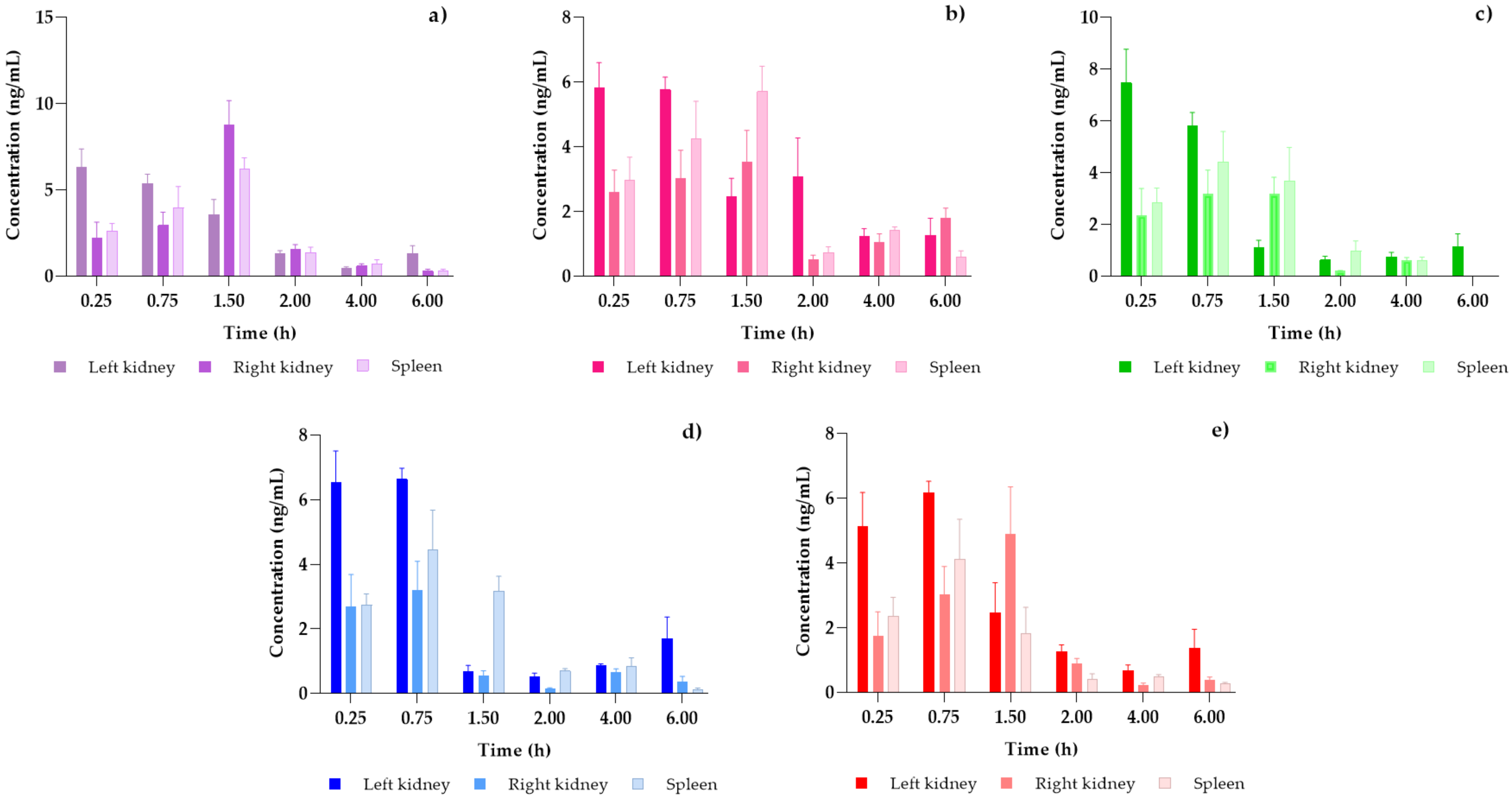

Figure S1. Tissue distribution in kidneys and spleen of 7-O-prenylscopoletin (PE), scoparone (SC), dimethylfraxetin (DF), herniarin (HR), 7-O-prenylumbelliferone (PU) after an oral dose administration of hexanic extract of *Tagetes lucida.* Values are presented as mean ± SEM (n = 5).
